# Alteration of gene expression profile following *PPP2R5C* knockdown may be associated with proliferation suppression and increased apoptosis of K562 cells

**DOI:** 10.1186/s13045-015-0125-5

**Published:** 2015-04-12

**Authors:** Sichu Liu, Qi Shen, Yu Chen, Chengwu Zeng, Changshu Cao, Lijian Yang, Shaohua Chen, Xiuli Wu, Bo Li, Yangqiu Li

**Affiliations:** Institute of Hematology, Jinan University, Guangzhou, 510632 China; Department of Hematology, The Second Clinical Medical college (Shenzhen People’s Hospital), Jinan University, Shenzhen, 518020 China; Key Laboratory for Regenerative Medicine of Ministry of Education, Jinan University, Guangzhou, 510632 China

**Keywords:** *PPP2R5C*, CML, BCR-ABL, Gene expression profile

## Abstract

We reported that knockdown of *PPP2R5C* by siRNA led to proliferation inhibition and apoptosis induction in K562 cells. In this study, we further characterized the gene expression profiles after *PPP2R5C* suppression by microarray analysis. Genes which participate in the MAPK, PI3K/AKT, and JAK/STAT pathways, were mainly altered in the K562 cells. We propose that the mechanism for proliferation inhibition and increased apoptosis of K562 cells following *PPP2R5C* suppression may be related to the alteration of expression profiles of *BRAF, AKT2, AKT3, NFKB2* and *STAT3* genes.

**Electronic supplementary material:**

The online version of this article (doi:10.1186/s13045-015-0125-5) contains supplementary material, which is available to authorized users.

## Findings

Overexpression of *PPP2R5C* is associated with the malignant transformation of several kinds of leukemia [1]. Recently we characterized the effects of downregulating *PPP2R5C* on the proliferation and apoptosis of K562 and Jurkat cells using different siRNAs which were targeting *PPP2R5C*. Significant proliferation inhibition was confirmed both in K562 and Jurkat cells, whereas apoptosis induction could only be observed in K562 and K562R cells [2,3].

**Figure 1:**
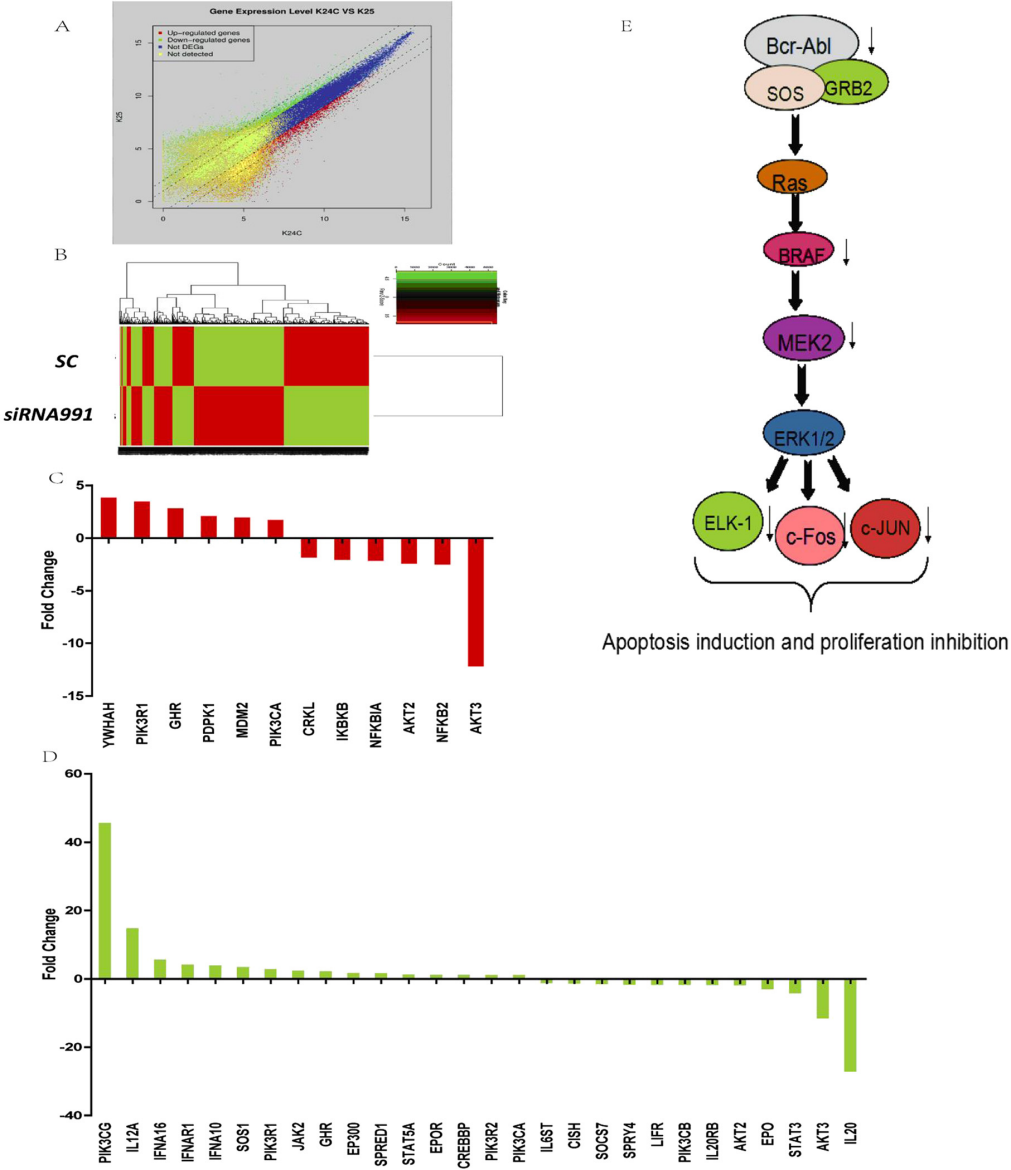
Microarray analysis for gene expression profiles of K562 cells after transfection with PPP2R5C-siRNA991. (**A**) Scatter plots comparing the gene expression profiles of siRNA991 and scrambled control (SC) transfected cells. The yellow dots represent genes undetected in both samples, blue dots represent genes present in both samples, red dots represent upregulated genes, and green dots represent downregulated genes. (**B**) The Affymetrix data were clustered, and the red and green colors represent the expression levels increased or decreased, respectively, with respect to the average expression across all samples. (**C**) PI3K/AKT signaling pathway genes differentially expressed in K562 cells after *PPP2R5C* suppression. (**D**) JAK/STAT signaling pathways genes differentially expressed in K562 cells after *PPP2R5C* suppression. (**E**) Schematic model of the BCR-ABL-mediated BRAF-MEK-FOS-JUN signaling pathway due to PPP2R5C suppression in K562 cells (modified from reference [8]).

To further investigate the gene expression profile, *PPP2R5C-*siRNA991-treated K562 cells were collected at 48 h post transfection when *PPP2R5C* mRNA was most suppressed [2]. Gene expression profiles were determined and analyzed by Affymetrix microarrays as reported (See Additional file 1 for methods and materials) [3,4]. Overall, 2,586 genes were upregulated and 2,601 genes were downregulated at least two-fold, when *PPP2R5C*-siRNA991 and SC-treated expression data were compared. We also found both the *Bcr* and *Abl* genes were downregulated (fold change: −1.23 and −1.53, respectively), suggesting that *PPP2R5C* is closely related to the BCR-ABL-mediated pathway. Besides that, there were changes in genes involved in different signaling pathways closely related to cell proliferation and apoptosis (Table 1, Figure 1A and B).

**Table 1:** **Cell proliferation and apoptosis genes altered after** ***PPP2R5C*** **knockdown in K562 cells in microarray analysis**

**Gene symbol**	**NCBI accession**	**Fold change**	**Description**	**Pathway**
BRAF	NM_004333	−2.24	v-raf murine sarcoma viral oncogene homolog B1	MAPK signaling pathway
MAP2K2	NM_030662	−2.39	mitogen-activated protein kinase kinase 2	MAPK signaling pathway
ELK1	NM_001114123	−2.65	ELK1, member of ETS oncogene family	MAPK signaling pathway
FOS	NM_005252	−3.12	FBJ murine osteosarcoma viral oncogene homolog	MAPK signaling pathway
JUN	NM_002228	−4.88	jun proto-oncogene	MAPK signaling pathway
NFKB2	NM_001077493 MAPK signaling pathway/AKT signaling pathway	−2.81	nuclear factor of kappa light polypeptide gene enhancer in B-cells 2 (p49/p100)	
AKT2	NM_001626	−2.72	V-akt murine thymoma viral oncogene homolog 2	MAPK signaling pathway/AKT signaling pathway
AKT3	NM_005465	−12.47	v-akt murine thymoma viral oncogene homolog 3 (protein kinase B, gamma)	MAPK signaling pathway/AKT signaling pathway
CRKL	NM_005207	−2.14	v-crk sarcoma virus CT10 oncogene homolog (avian)-like	MAPK signaling pathway/AKT signaling pathway
IL6ST	NM_001190981	−2.13	interleukin 6 signal transducer (gp130, oncostatin M receptor)	Jak-STAT signaling pathway
STAT3	NM_003150 Jak-STAT signaling pathway	−5.08	signal transducer and activator of transcription 3 (acute-phase response factor)	
MDM2	NM_002392	2.26	Mdm2 p53 binding protein homolog	AKT Signaling Pathway/p53Signaling Pathway
ATM	NM_000051	−2.30	ataxia telangiectasia mutated	p53Signaling Pathway

Aberrant BCR-ABL tyrosine kinase activity plays a crucial role in the pathogenesis of CML [5,6]. Moreover, abnormal interactions between the BCR-ABL oncoprotein and other molecules lead to the disruption of the major cellular processes, including the MAPK, JAK/STAT and PI3K/AKT signaling pathway, which can result in the dysregulation of proliferation and apoptosis [7].

In the MAPK signaling pathway, 67 genes were differentially expressed including 20 upregulated and 47 downregulated genes. The significantly downregulated genes including *BRAF, MAP2K2, ELK1*, *NFKB2, FOS,* and *JUN*. Downregulated *BRAF* might decrease the expression and phosphorylation of the downstream proteins MAP2K2, ELK1, NFKB2, FOS and JUN (Figure 1C) [8]. As a consequence, the major effects of the proliferation inhibition in *PPP2R5C-*siRNA991-treated K562 cells might be via the BRAF inhibition.

There were alterations involved in the PI3K/AKT signaling pathway including 6 upregulated and 6 downregulated genes (Figure 1D). *PPP2R5C* suppression predominantly resulted in *MDM2* upregulation and downregulation of *CRKL*, *AKT2*, *AKT3*, and *NFKB2*. PI3K activates AKT kinases and causes the phosphorylation of downstream factors that regulate the AKT-mediated cellular apoptotic machinery [8,9], while downregulation of *CRKL* weakens BCR-ABL binding to PI3K, leading to reduced AKT phosphorylation. Moreover, a reduction in NFKB2 might be directly linked to the induction of apoptosis [10], and MDM2, a negative regulator of p53, might indirectly affect apoptosis [11]. Therefore, it is thought that AKT2, AKT3 and NFKB2 might be involved in apoptosis induction in K562 cells after *PPP2R5C* inhibition.

In the JAK/STAT signaling pathway, 28 genes were differentially expressed, including 16 upregulated and 12 downregulated genes (Figure 1E). The downregulated genes *IL6ST* and *STAT3* may play important roles in cell proliferation through inhibition of the IL-6/JAK/STAT3 pathway, and STAT3, which is a signal transducer, plays a key role in cell survival in human hematopoietic malignancies [12]. Thus, *PPP2R5C* suppression might have effect on the JAK/STAT pathway through *STAT3* downregulation, leading to proliferation inhibition in K562 cells.

Because the mediation of cell proliferation, differentiation, and transformation functions of *PPP2R5C* is based on its induction of p53 dephosphorylation at various residues [13,14], a dominant alteration in p53 pathway was found for *ATM*, which had 2.3-fold downregulation, and *MDM2*, which was upregulated 2.26-fold. These results are similar to our previous finding in Jurkat cells in which we showed that proliferation was suppressed by *PPP2R5C*-siRNA. It is thought that ATM downregulation and MDM2 upregulation might lead to a decreased transcriptional activation level for p53, suggesting that the *PPP2R5C*-mediated p53 function might use the same signaling pathway in different leukemia cells.

In conclusion, we characterized altered expression profile of genes related to the BCR-ABL signaling pathway in *PPP2R5C*-siRNA-treated K562 cells. The mechanism of *PPP2R5C-*suppression-mediated inhibition of proliferation and increased apoptosis in K562 cells may be related to the MAPK, PI3K/AKT, JAK/STAT pathways through *BRAF, AKT2, AKT3, NFKB2* and *STAT3* downregulation. However, further validation of the altered genes and related proteins is needed.

## Additional file

Additional file 1:
**Methods and materials.**
