# Supplementary material for: Alteration of gene expression profile following PPP2R5C knockdown may be associated with proliferation suppression and increased apoptosis of K562 cells
Source: J Hematol Oncol. 2015 Apr 12;8:34. doi: 10.1186/s13045-015-0125-5 (PMC4414434; doi:10.1186/s13045-015-0125-5)
Supplement: Additional file 1: — Methods and materials. [file 13045_2015_125_MOESM1_ESM.docx]

**Methods and materials**

***Cell culture***

The human chronic myelogenous leukemia cell line K562 (Institutes for Biological Sciences Cell Resource Center, Chinese Academy of Sciences, Shanghai, China) was grown in the complete Roswell Park Memorial Institute (RPMI) 1640 medium (Gibco-BRL) with 10% fetal calf serum (Sijiqing Co.) in a 95% humidified incubator at 37℃ and 5% CO_2_.

***PPP2R5C-siRNA and delivery***

*PPP2R5C*-siRNA991 (Chinese patent number: ZL 201110337837.1), which targets a domain between the eighth and ninth exons in the *PPP2R5C* gene (ACCESSION NM_178587), and a scrambled nonsilencing siRNA control (SC) were designed with online software (www.invitrogen.com) and synthesized by Invitrogen [2,3].

The siRNA was delivered using the nucleofection method. K562 cells were resuspended at 2.5 x 10^6^ per 100 μL of the appropriate Nucleofector^TM^ kit V solution (Amaxa Biosystems) , and they were nucleofected with 3 μg of *PPP2R5C*-siRNAs or a control nonsilencing scrambled (SC) siRNA using the T-003 program of the Nucleofection Device II (Amaxa Biosystems). Mock-transfected cells nucleofected without siRNA were used as a negative control. After nucleofection, the cells were immediately mixed with 500 μL of pre-warmed culture medium and transferred into culture plates. The treated cells were incubated at 37℃ and collected for RNA isolation.

***RNA isolation and Microarray analysis***

According to our previous study [2], *PPP2R5C* siRNA991(Chinese patent number: ZL 201110337837.1)-treated K562 cells were collected at 48 h post transfection when *PPP2R5C* mRNA was most suppressed. RNA was isolated using TRIzol (Invitrogen). Total RNA (> 3 μg) was processed for global gene expression profile analysis using the Affymetrix HG-U133 Plus 2.0 array (Shanghai Biochip Co. Ltd). Affymetrix microarray analysis was performed using Gene Spring GX11.0 software (Agilent Technologies)[3-4]. Probe sets displaying a signal log ratio indicating an increase or marginal increase i.e., log ratio ≧1(n), and detection of an experimental group displaying a signal change with P represented upregulated genes. Conversely, probe sets displaying a signal log ratio indicating a decrease or marginal decrease i.e., log ratio ≦ - 1(n), and detection of a control group displaying a signal change with P represented downregulated genes. The resulting data were analyzed using the SBC Analysis System. After normalization and correction, the log2 fluorescence intensity value for each gene was obtained [3,4]. A two-fold minimum difference was considered significant. The fold change for all genes was calculated by comparing the *PPP2R5C*-siRNA991- and SC-treated K562 cells.
